# Expression of Plet1 controls interstitial migration of murine small intestinal dendritic cells

**DOI:** 10.1002/eji.201847671

**Published:** 2018-12-14

**Authors:** Julien J. Karrich, Mónica Romera‐Hernández, Natalie Papazian, Sharon Veenbergen, Ferry Cornelissen, Patricia Aparicio‐Domingo, Frances H. Stenhouse, C. Diana Peddie, Remco M. Hoogenboezem, Chelsea W. J. den Hollander, Terri Gaskell, Tanya Medley, Louis Boon, C. Clare Blackburn, David R. Withers, Janneke N. Samsom, Tom Cupedo

**Affiliations:** ^1^ Department of Hematology Erasmus University Medical Center Rotterdam The Netherlands; ^2^ Laboratory of Pediatrics Division of Gastroenterology and Nutrition Erasmus University Medical Center Rotterdam The Netherlands; ^3^ MRC Centre for Regenerative Medicine University of Edinburgh Edinburgh UK; ^4^ Bioceros B.V. Utrecht The Netherlands; ^5^ MRC Centre for Immune Regulation University of Birmingham Birmingham UK

**Keywords:** CD103, Intestinal dendritic cells, Migration, Plet1, Wound healing

## Abstract

Under homeostatic conditions, dendritic cells (DCs) continuously patrol the intestinal lamina propria. Upon antigen encounter, DCs initiate C‐C motif chemokine receptor 7 (CCR7) expression and migrate into lymph nodes to direct T cell activation and differentiation. The mechanistic underpinnings of DC migration from the tissues to lymph nodes have been largely elucidated, contributing greatly to our understanding of DC functionality and intestinal immunity. In contrast, the molecular mechanisms allowing DCs to efficiently migrate through the complex extracellular matrix of the intestinal lamina propria prior to antigen encounter are still incompletely understood. Here we show that small intestinal murine CD11b^+^CD103^+^ DCs express Placenta‐expressed transcript 1 (Plet1), a glycophoshatidylinositol (GPI)‐anchored surface protein involved in migration of keratinocytes during wound healing. In the absence of Plet1, CD11b^+^CD103^+^ DCs display aberrant migratory behavior, and accumulate in the small intestine, independent of CCR7 responsiveness. RNA‐sequencing indicated involvement of Plet1 in extracellular matrix‐interactiveness, and subsequent in‐vitro migration assays revealed that Plet1 augments the ability of DCs to migrate through extracellular matrix containing environments. In conclusion, our findings reveal that expression of Plet1 facilitates homeostatic interstitial migration of small intestinal DCs.

## Introduction

Most cells in the mammalian body are contained within a singular environment throughout their life span, with movement generally restricted to a microenvironment to which cells have optimally adapted. This is in sharp contrasts to immune cells, that migrate through highly divergent surroundings, ranging from the fluidics of blood and lymph to the cell and matrix‐dense environments of peripheral organs 1. The quintessential example of migrating immune cells are dendritic cells (DCs). DCs patrol peripheral tissues, efficiently navigating the extracellular matrix (ECM) with which they are continuously interacting. Upon antigen encounter, DCs become untethered from the ECM, gain the ability to egress from peripheral tissues, enter the fluidic environment of the lymph 2, 3, 4 and migrate to the draining lymph nodes (LN) in a CCR7‐dependent manner 5, 6. A number of mechanisms and molecules that allow tissue egress and entry into lymph vessels upon DC maturation have been clarified in recent years 5, 7. In contrast, the mechanistic underpinnings that allow immature DCs to efficiently surveil peripheral tissues are largely unknown.

In order to accommodate efficient tissue egress and entry into the draining lymphatics, activatied DCs in barrier tissues alter their migratory machinery. In the skin, DC activation leads to functional deactivation of cell adhesion and integrin receptors, allowing for detachment from the ECM 8, 9. Similarly, intestinal DC activation leads to migration and tissue egress by mechanisms independent of integrin interactions but driven by amoeboid‐like movement through the ECM 10.

There is evidence to suggest that microbiota‐derived signals modulate homeostatic DC behaviour. Intestinal bacteria can actively induce migration of CD103^+^ DCs into the intestinal epithelial barrier 11, 12 and in Toll‐like receptor (TLR)‐signalling deficient Myd88^−/−^ mice, migration of CD103^+^ intestinal DCs to gut‐draining LN is aberrant 13.

Based on such associations between microbial signals and interstitial DC positioning, we hypothesized that sensing of microbiota‐derived signals influences motility of immature DCs within mucosal tissues. In this study, we identified expression of Placenta‐expressed transcript 1 (Plet1), a surface protein involved in keratinocyte migration 14, on intestinal migratory DCs. Plet1 is an orphan Glycophoshatidylinositol (GPI)‐anchored surface protein first identified as a protein recognized by the monoclonal antibodies (mAbs) MTS20 and MTS24 15 and expressed on various cells and organs, such as the early thymic epithelial cells 16, mouse hair follicular keratinocyte progenitor cells 17, and major duct epithelium 15, 18.

Using gene‐targeted mice, unbiased transcriptomic profiling and in vitro assays we show that Plet1 allows DCs to efficiently migrate through ECM‐rich environments, independent of CCR7 ligation. Our work links microbial presence to homeostatic DC functionality, and identifies a protein used by the small intestinal immune system to modulate steady state activity of sentinel dendritic cells in response to changing environments.

## Results

### Plet1 marks migratory mucosal DCs and is regulated by microbiota

**Figure 1:**
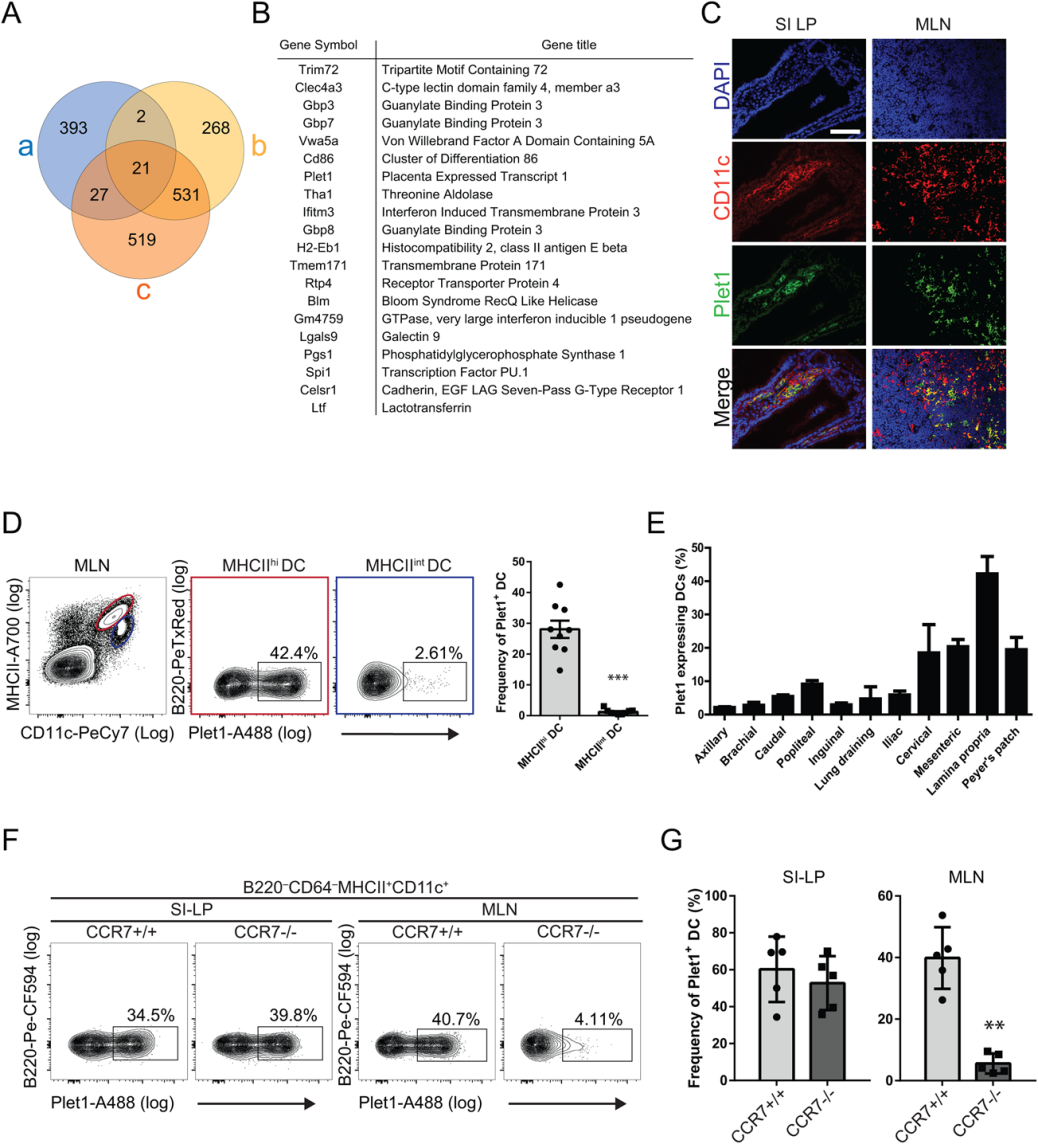
Plet1 is expressed on mucosal migratory DC and regulated by microbiota. (A) Venn diagram showing in‐silico combinatorial transcriptome analysis of differentially expressed genes between CD103^+^CD11b^+^ DC and CD103^+^CD11b^−^ DC (a, blue circle; http://www.immgen.org), and ileum of germ free mice 4 days (b, yellow circle) and 30 days after conventionalization (c, orange circle; from: 19). (B) List of genes differentially expressed in a, b, and c. (C) Immunofluorescence analysis of small intestinal tissue (SI‐LP, left panels), and MLN (right panels) showing expression of Plet1 (green) and CD11c (red). Plet1^+^CD11c^+^ cells appear yellow (scale bar 200 μm). Images are from a single animal representative of 3 independently analyzed mice. (D) Representative flow cytometric analysis showing percentages of Plet1^+^ cells within MHCII^hi^ migratory DC (red) and MHCII^int^ resident DC (blue) isolated from MLN (for DC gating strategy see Supporting Information Fig. 1). Data are shown as mean + SEM and are pooled from 2 independent experiments, 4–5 mice per experiment. Unpaired Mann‐Whitney test, ^***^ *p* < 0.001. (E) Frequency of Plet1‐expressing CD11c^+^ DC. Data are shown as mean +SEM and are pooled from two independent experiments, 2–4 mice per experiment. (F) Representative flow cytometry analysis of Plet1 expression on DC in small intestinal lamina propria and MLN of CCR7^−/−^ mice and controls. (G) Frequency of Plet1‐expressing DC in the lamina propria and MLN of CCR7^−/−^ animals, compared to littermate controls. Data are shown as mean +SEM and are pooled from two independent experiments, 2–3 mice per experiment. Unpaired Mann–Whitney test, ^**^ *p* < 0.01.

Homeostatic interstitial migration of small intestinal DCs is likely influenced by signals derived from the intestinal microbiota. To identify microbiota‐modulated DC‐expressed molecules we performed combinatorial in‐silico analysis of publicly available microarray datasets. We selected genes whose transcription was upregulated in small intestinal tissue of germ‐free mice at 4 or 30 days after conventionalization with total fecal microbial community (GSE32513) 19 and which were overexpressed in intestinal migratory CD11b^+^CD103^+^ DCs compared to CD11b^−^CD103^+^ DCs (GSE15907) 20. This identified 20 genes (Fig. 1A and B) 3 of which (Plet1, Lgals9, and Celsr1) had previously been associated with cell migration or cell adhesion (Supporting Information Table 2). Interestingly, Plet1 (also known as MTS24 15 or AgK114 21, 22) has an important function in keratinocyte migration during wound healing 14 and Plet1 expression on small intestinal CD45^+^ hematopoietic cells was previously noted 15. We therefore analyzed Plet1 protein expression on mononuclear phagocytes in mouse small intestine. Histological analyses revealed Plet1 expression on CD11c^+^ cells in small intestine lamina propria (SI‐LP), and in the mesenteric lymph nodes (MLN) draining the small intestine (Fig. 1C). The presence of CD11c^+^Plet1^+^ cells in both intestine and gut‐draining lymph nodes suggested that Plet1 could be expressed on a migrating DC population. We therefore used a more elaborate labeling to identify intestinal DCs by flow cytometry (gating in Supporting Information Fig. 1, according to refs 23, 24). This revealed that Plet1 was expressed on CD11c^+^MHCII^+^ DCs, and absent from cells expressing CD64, including monocytes and macrophages (Supporting Information Fig. 2)^23^. Moreover, nearly all Plet1^+^ DCs were contained within the MHCII^hi^ migratory DC population (Fig. 1D). In line with this expression on migrating DCs, the frequency of Plet1‐expressing cells within mucosal LNs was consistently higher compared to peripheral LNs (Fig. 1E). Plet1 transcripts and protein were undetectable in splenic DCs (data not shown). Finally, to prove that Plet1 is expressed on DCs migrating from the intestine to the draining LNs under homeostatic conditions we analyzed the presence of Plet1^+^ DCs in CCR7^−/−^ animals, where migratory DCs fail to egress from the SI‐LP 5. While the frequency of Plet1^+^ DCs in the SI‐LP of CCR7^−/−^ animals was similar to that of littermate control mice, CCR7^−/−^ MLN were devoid of Plet1‐expressing DCs (Fig. 1F and G). Together, these data identify the protein Plet1 as a marker for small intestinal migratory DCs under steady state conditions.

### CD11b^+^CD103^+^ DCs express Plet1 prior to intestinal egress

**Figure 2:**
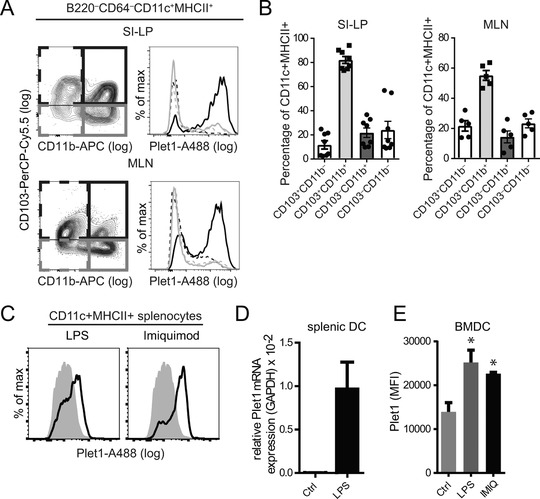
Plet1 is expressed by intestinal CD11b^+^CD103^+^ DC. (A) Distribution of Plet1 on small intestinal lamina propria (LP)‐, and MLN‐DC populations was analyzed by flow cytometry according to expression of CD103 and CD11b (CD103^−^CD11b^−^, dotted grey line; CD103^+^CD11b^−^, dotted black line; CD103^−^CD11b^+^, gray line; CD103^+^CD11b^+^, black line) (Gating as in Supporting Information Fig. 1). (B) Frequency of Plet1+ cells within DC subsets according to CD103 and CD11b in the SI‐LP (left panel) and in the MLN (right panel), as shown in (A) For A and B: pooled from 2–3 independent experiments, 2–4 mice per group, mean + SEM. (C) Flow cytometric assessment of Plet1 expression on ex‐vivo splenic CD11c+ DC stimulated with LPS (left panel, black line), or with Imiquimod (right panel, black line), as compared to unstimulated cells (grey filled histograms) pooled from 2 independent experiments, 3–4 mice per group. (D) Relative expression of *Plet1* transcripts in ex‐vivo splenic DC, cultured overnight with or without LPS. Pooled from 2 independent experiments, 1–2 mice per group and showing mean + SEM. (E) MFI of Plet1 expression on BMDC assessed by flow cytometry. 3 animals analyzed in a single experiment. (Unpaired Mann–Whitney test, ^*^ *p* < 0.05, mean + SEM).

Several small intestinal DC subsets have the ability to migrate to the draining LNs under homeostasis 25. Flow cytometry revealed that Plet1 was mainly expressed by CD11b^+^CD103^+^ DCs, with approximately 80% of these cells expressing Plet1 in the SI‐LP and approximately 60% in MLN (Fig. 2A and B). Since our in‐silico analysis suggested that Plet1 transcription was regulated by microbial signals, we stimulated bone marrow‐derived DCs (BMDCs) and freshly isolated murine splenic DCs for 24 h with ligands for Toll‐like receptors (TLR)‐4 and TLR7. Both ligands increased expression of Plet1 on ex‐vivo splenic DCs (Fig. 2C and D). In addition, TLR4 stimulation induced *Plet1* mRNA expression on in‐vitro generated BMDC (Fig.e 2E). Collectively, these findings identify Plet1 as a TLR‐regulated surface protein preferentially expressed on CD11b^+^CD103^+^ small intestinal DCs prior to exit from the lamina propria.

### Normal intestinal immune system development in Plet1^−/−^ mice

**Figure 3:**
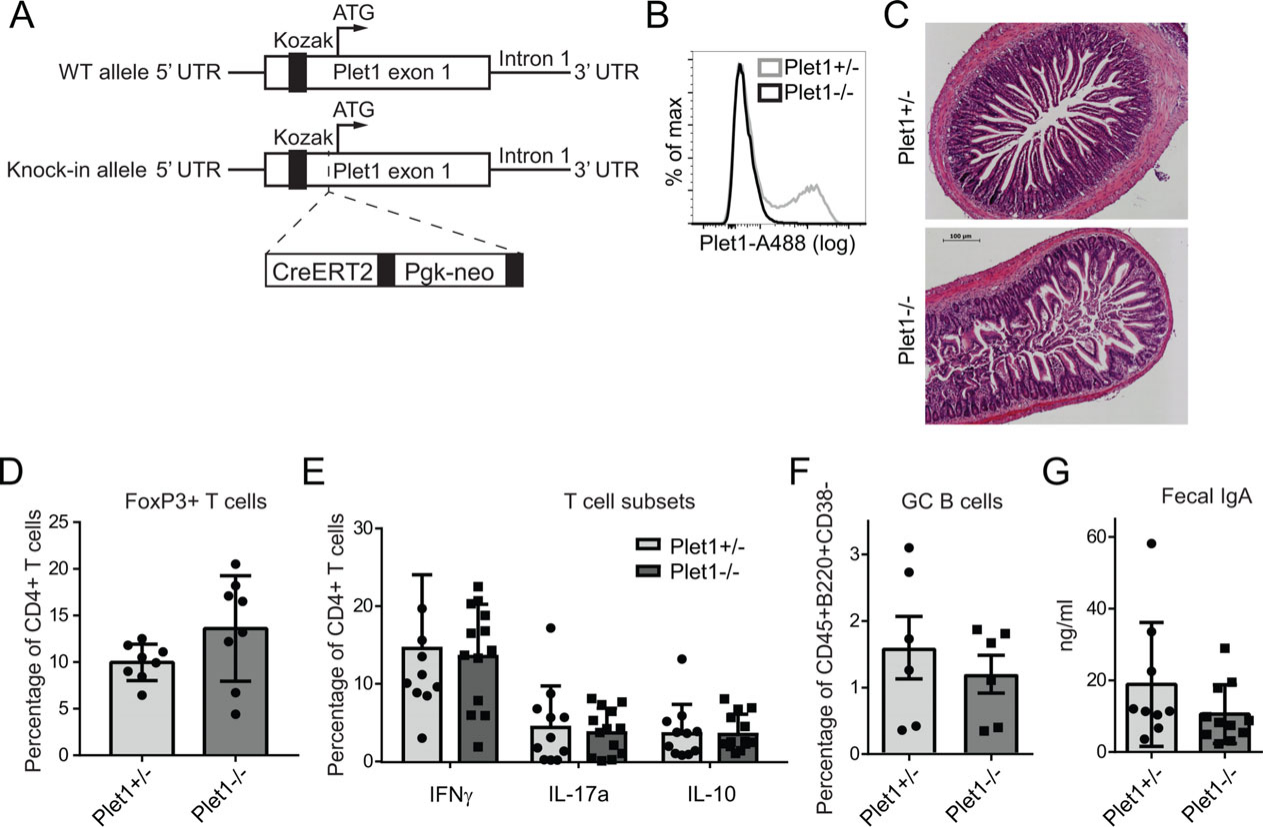
Normal intestinal immune system development in Plet1^−/−^ mice. (A) Schematic representation of the targeting strategy for knock in‐mediated silencing of Plet1. (B) Plet1 expression on small intestinal DC isolated from Plet1^+/−^ (gray line), or Plet1^−/−^ (black line) mice was analyzed by flow cytometry. >10 mice per genotype (C) Representative H&E staining of duodenal sections from Plet1^+/−^ or Plet1^−/−^ animals. 4–5 mice per genotype from 2 independent experiments, scale bar 100 μm. (D) Frequency of FoxP3^+^ T cells within total SI‐LP CD4^+^ T cells from Plet1^+/−^ or Plet1^−/−^ animals. 2 independent experiments, 3 mice per group. (E) Frequency of IFNγ^+^, IL‐17^+^, and IL‐10^+^ producing CD4^+^ MLN T cells following restimulation for 4 h with PMA/Ionomycin. Pooled data from 2 independent experiments with 4 mice per group. (Unpaired Mann–Whitney test, mean + SEM) (F) Mean frequencies of CD38^−^ germinal center (GC) B cells isolated from Plet1^+/−^, or Plet1^−/−^ Peyer's patches. Pooled data from 2 independent experiments with 3 mice per group. (Unpaired Mann‐Whitney test, mean + SEM) (G) Quantification of IgA within total fecal content of colons from Plet1^−/−^ and littermate controls, normalized to weight. Pooled data from 2 independent experiments with 4–5 mice per group. (Unpaired Mann‐Whitney test, mean + SEM).

To investigate the function of Plet1 in‐vivo we generated Plet1 knock‐out mice by replacing part of exon 1 of the *Plet1* gene with a CreERT2‐containing targeting cassette (Fig. 3A). Unfortunately, none of the founder strains with disturbed Plet1 transcription showed transcription of the CreERT2 insert, precluding the use of the Cre recombinase, effectively generating a conventional knock‐out strain (Fig. 3B). Plet1^−/−^ mice were viable, born at Mendelian ratios and histological examination of the small intestine of 8 to 12 week old animals did not reveal any signs of spontaneous pathology (Fig. 3C). To assess whether Plet1 deficiency affects differentiation of T helper cell subsets we analyzed composition of the SI‐LP T cell pool. Frequencies of Foxp3 expressing regulatory T cells (Fig. 3D), or T cells secreting IFNγ, IL‐17A or IL‐10 (Fig. 3E) were similar in Plet1^−/−^ mice compared to littermate controls. To determine possible impact on B cell immunity we enumerated germinal center B cells in Peyer's patches as well as fecal IgA content (Fig. 3F and G). Again no differences were observed between Plet1^−/−^ mice and littermate controls. Together this shows that Plet1 deficiency does not affect intestinal immune cell composition and does not lead to spontaneous intestinal pathology.

### Plet1 alters steady state distribution of migratory CD11b^+^CD103^+^ DCs in the small intestine

**Figure 4:**
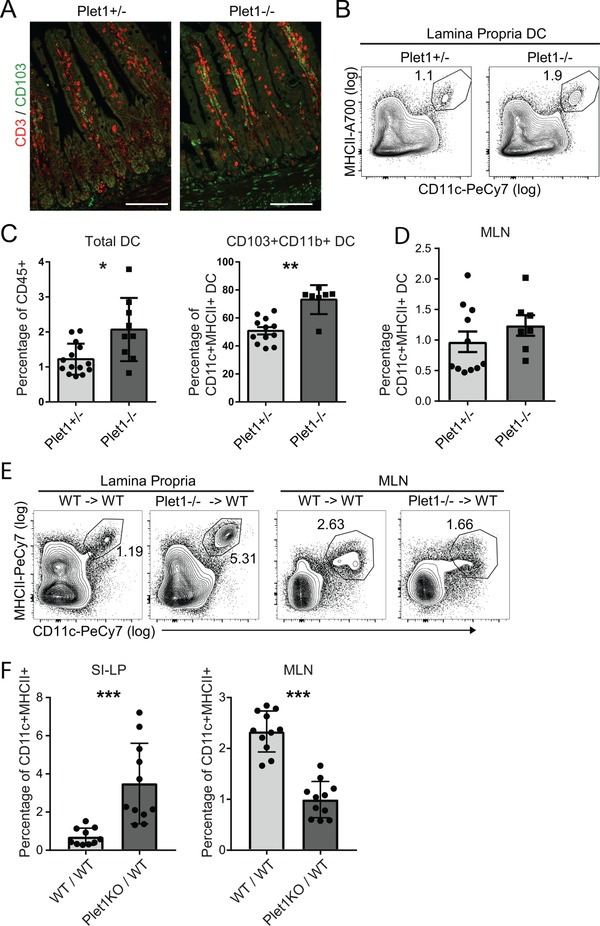
Plet1 deficiency alters homeostatic localization of intestinal CD11b^+^CD103^+^ DC. (A) Representative histology of illeal sections of Plet1^+/−^ and Plet1^−/−^ mice. CD3 in red, CD103 in green. Scale bar: 100μm. Images from a single experiment representative of 2 experiments with 1–2 mice per experiment (B) flow cytometry plots showing frequency of total DC in SI‐LP of Plet1^+/−^, or Plet1^−/−^ animals. (C) Frequency of total DC in small intestinal lamina propria and (D) frequency of CD11b^+^CD103^+^ DC in small intestinal lamina propria. Pooled data of 2 independent experiments, 4–7 mice per group. Unpaired Mann–Whitney test, ^*^ *p* < 0.05, ^**^ *p* <0.005, mean + SEM. (E) Representative plots of SI‐LP and MLN cells isolated from WT or Plet1^−/−^ bone marrow chimeric mice. (F) Frequency of total CD11c^+^MHCII^+^ DC in SI‐LP (left panel), and MLN (right panel) of WT or Plet1^−/−^ bone marrow chimeric mice. Pooled data from 2 independent experiments, 5–6 mice per group. Unpaired Mann–Whitney test, ^***^ *p* < 0.001, mean + SEM).

To define whether expression of Plet1 influences homeostatic localization of DC, we analyzed CD103‐expressing SI‐LP cells in Plet1^−/−^ and control mice. Histological analyses of illeal sections suggested an increase in CD103^+^CD3^−^ cells within the lower parts of the villi in Plet1^−/−^ mice (Fig. 4A). To quantify this difference, we analyzed total SI‐LP by flow cytometry. Absence of Plet1 resulted in a significant increase in the percentage of total intestinal DCs (Fig. 4B and C) and this increase was mainly due to an accumulation of CD11b^+^CD103^+^ DCs (Fig. 4C). Frequencies of DCs in the MLN were comparable between the two groups (Fig. 4D). To address whether this altered phenotype was DC intrinsic, and to exclude any possible confounding effects of Plet1 absence on tubular epithelial cells 21, we generated bone marrow chimeric mice using either Plet1^−/−^ or WT bone marrow. Analyses of DC subsets revealed that hematopoietic deficiency of Plet1 resulted in an increased frequency of total DCs in the SI‐LP, that was concomitant with a diminished proportion of total DCs in the MLN (Fig. 4E and F). Taken together, this indicates that Plet1‐deficiency alters homeostatic positioning of small intestinal DCs.

### Plet1 controls in‐vitro DC migration through 3D environments

**Figure 5:**
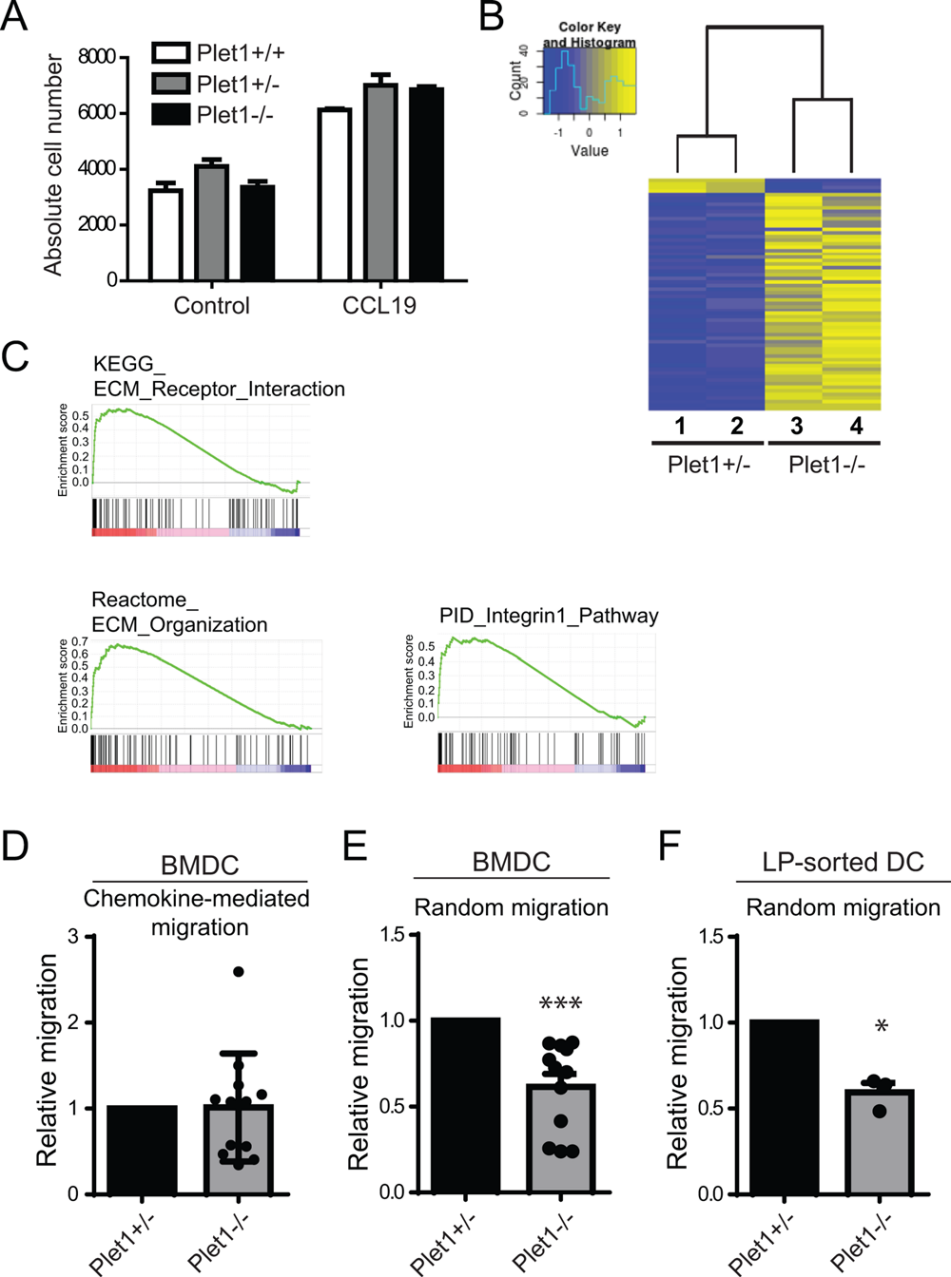
Plet1 deficiency alters migration of DC in 3D collagen environments in‐vitro. (A) Absolute cell numbers of migrated cells in response to CCL19 using BMDC differentiated from Plet1^−/−^ or littermate controls animals. Pooled data from 2 independent experiments, 1 mouse and 2 experimental repeats per experiment. (Unpaired Mann‐Whitney test, mean + SEM) (B) Heat map showing significantly different genes between CD11b^+^CD103^+^ DC isolated from Plet1‐sufficient and Plet1‐deficient mice, by RNA sequencing analysis. Single experiment with 2 animals per genotype (C) Gene Set Enrichment Analysis highlighting a putative role for Plet1 in regulating interaction with the extracellular matrix. (D) CCL19‐dependent migration of Plet1^−/−^ or control BMDC loaded in Collagen I matrix. (E) Chemokine independent migration of Plet1^−/−^ or control BMDC loaded in Collagen I matrix. (Pooled data from 3 independent experiments using independent BMDC cultures and 4 experimental repeats. Unpaired Mann–Whitney test, ^***^ *p* < 0.001) (F) Chemokine independent migration of freshly isolated SI‐LP DC loaded in Collagen I matrix. (Pooled data from 2 independent experiments with a single mouse and 4 experimental repeats per experiments. Unpaired Mann–Whitney test, ^*^ *p* < 0.05).

The accumulation of intestinal CD11b^+^CD103^+^ DCs in the absence of Plet1 could result from altered responsiveness to CCR7 ligands and subsequent reduced migration to the MLN. To directly assess CCR7 responsiveness, we generated BMDCs from Plet1^−/−^ and littermate control bone marrow. Notably, BMDCs express Plet1 (Fig. 2F), making this a suitable model to study functional consequences of Plet1 expression. Generation and activation of Plet1^−/−^ BMDC was similar to controls (Supporting Information Fig. 3A–D). The ability of BMDCs to migrate in a CCR7‐dependent manner was tested in trans‐well assays. After 2 h, the number of migrated BMDCs was comparable between Plet1^−/−^ and heterozygous or homozygous controls, indicating that absence of Plet1 does not reduce CCR7 responsiveness (Fig. 5A). We next took an unbiased approach to identify transcriptional changes affected by absence of Plet1 and compared FACS‐sorted CD11b^+^CD103^+^ small intestinal lamina propria DCs from Plet1^+/−^ and Plet1^−/−^ animals by RNA‐sequencing (Fig. 5B). In the absence of Plet1, 66 genes were significantly altered, with the majority of genes (62 genes) showing increased transcription in Plet1^−/−^ DCs (Supporting Information Table 3). This suggested that Plet1 is predominantly involved in constraining gene expression (Fig. 5B). Gene set enrichment analysis (GSEA) indicated enrichment of gene sets associated with cell‐cell interactions and interactions with the ECM, as well as integrin signaling in the absence of Plet1 (Fig. 5C). This led us to hypothesize that Plet1 limits DCs ‐ ECM interactions, allowing for efficient migration through complex 3D environments rich in ECM. To directly test this hypothesis we interrogated the ability of Plet1^−/−^ and control BMDCs to migrate through a 3D collagen I matrix, the major component of the small intestinal ECM in human and mouse 26, seeded in the upper well of a trans‐well chamber. In response to the CCR7 ligand CCL19, Plet1^−/−^ and Plet1^+/+^ BMDCs were equally capable of navigating out of the collagen matrix (Fig. 5D). Since homeostatic migration through tissues is CCR7 independent, we subsequently determined the ability of BMDCs to exit the collagen I matrix in the absence of CCR7 ligands. In this setup, Plet1^−/−^ DCs had a severe defect in migrating through the extracellular matrix, and compared to control BMDC, only half of the cells egressed from the collagen I matrix (Fig. 5E). Since BMDCs are likely not a faithful representation of intestinal DCs 27, we next isolated total SI‐LP DCs from Plet1^−/−^ and littermate control mice and seeded these into collagen I containing trans‐wells. Similar to BMDCs, Plet1‐deficient SI‐LP DCs directly ex‐vivo were blunted in their ability to migrate through a 3D collagen environment, again showing a 2 fold reduction in number of cells migrating out of the collagen gels (Fig. 5F). Altogether, our findings identify a role for Plet1 in facilitating DC movement through ECM‐rich 3D environments.

## Discussion

The mechanistic underpinnings of CCR7‐dependent migration of activated, maturing DCs out of the intestine and into the draining lymph nodes have been elucidated in detail 5, 7. In sharp contrast, the factors that influence movement of DCs through barrier tissues such as the intestine are much less defined. Here we identified the surface protein Plet1 as preferentially expressed on small intestinal CD11b^+^CD103^+^ migratory DCs, functionally endowing these cells with the ability to efficiently navigate ECM‐rich 3D environments ex‐vivo.

Plet1 has been implicated in cellular migration in different cell systems. During keratinocyte migration in in‐vitro and in‐vivo models of wound healing, Plet1 alters the adhesion of leading edge cells to Collagen type I and Collagen type IV 14, 22. In our hands, Plet1^−/−^ small‐intestinal DCs failed to migrate through Collagen type I ECM, revealing a previously unappreciated parallel between migration of DCs and keratinocytes. Our in‐vitro experiments were performed with isolated lamina propria DC as well as with GM‐CSF matured BMDC. It is important to realize that expression of CCR7 on BMDC is lower in GM‐CSF‐derived cultures compared to FLT3L‐matured BMDC 28. This might imply that the differences observed could be even more pronounced when using alternative methods of cell preparation.

Our data show that in the small intestinal immune system, Plet1 expression is enriched on MHCII^hi^ migratory DCs and that CD11b^+^CD103^+^ DCs are the main Plet1‐expressing migratory DC population in the steady‐state small intestine.

In the absence of Plet1, CD11b^+^CD103^+^ DCs accumulate in the intestinal lamina propria, even though these cells maintain functional CCR7 responses in‐vitro. An explanation for this accumulation might be in reduced motility of Plet1‐deficient CD11b^+^CD103^+^ DCs. A reduction in distance covered per time unit due to altered locomotion could increase the average time to antigen encounter and ensuing maturation with CCR7 induction. This would prolong dwell‐time of DCs in the small intestine and ultimately result in an accumulation of cells in this compartment.

The small‐intestinal adaptive immune system was unaltered in Plet1^−/−^ animals at steady state. However, our findings on Plet1 function do raise an intriguing possibility that warrants further investigation. Reduced motility of Plet1‐deficient DCs might not only alter the time to antigen encounter, it might also negatively affect the diversity of antigens encountered by individual DC. This would result from the possibility that if individual DCs cover less distance while patrolling the organ they might engulf antigens from a smaller area of the intestine. Whether this is indeed the case and whether this translates into altered or delayed immune responses under infectious or inflammatory conditions is of clear interest, but beyond the scope of the current study.

The exact molecular mechanism by which Plet1 modulates DC motility remain undefined. Plet1 is a molecule whose expression is very restricted. Notably, many of the Plet1 expressing cells are epithelial by nature and exhibit stem or progenitor cell characteristics. Plet1 is expressed by uterine luminal epithelium 29 and by mammary gland epithelium 30; furthermore, it demarcates very distinct populations of progenitor cells in hair follicles 14, 31, in pancreatic duct epithelium 15, and in the thymus 15, 16. Interestingly, both epithelial cells and progenitor cells critically depend on interactions with ECM for their differentiation, survival and adequate positioning 32. It is therefore tempting to speculate that Plet1 may be involved in regulating integrin–ECM interactions. Our RNA‐sequencing analysis revealed pathways involved in interactions with the ECM, in line with previous data on keratinocyte migration. Moreover, in‐silico 3D structure prediction of Plet1 using web‐based prediction software (Phyre2)^33^, reveals a high structural homology with the N‐terminal domain of the secreted glycoprotein Reelin (Reelin‐n; Supporting Information Fig. 4)^34^, ^35^, that is required for migration, differentiation, and proliferation of neural stem cells in the brain 36, 37. According to the Immgen datasets 38, expression of Plet1 is not restricted to intestinal DCs. It is also transcribed by skin epidermal/Langerhans cells and lung CD103^+^ DCs. This suggests that Plet1‐controlled tissue migration could be a more common feature of mucosal DCs, including skin and lung DCs, rather than being specific for the small intestine.

In humans, *Plet1* is poorly transcribed in epithelial cells 29, yet a role for ECM‐associated proteins in affecting human DC function and migration exists 39. Additional work is needed to address Plet1 expression in the human intestinal immune system, or to identify possible alternative molecules that can actively modulate ECM interactions to allow cellular motility within tissues.

Summarizing, we have identified Plet1 as a novel marker for migrating CD11b^+^CD103^+^ small‐intestinal DCs, regulated by TLR signaling. Plet1 enhances DC motility in ECM‐rich 3D environments and we propose that the intestinal innate immune system uses expression of Plet1 to actively modulate its ability to scan intestinal tissue for antigen.

## Materials and methods

### Mice

C57BL/6J wild‐type (WT) mice were bred and maintained at the Erasmus University Medical Center (Rotterdam, The Netherlands). CCR7^−/−^ mice were bred and maintained at the Biomedical Services Unit at the University of Birmingham. Plet1^−/−^ mice were generated at the University of Edinburgh by inserting a CreErt2 construct into exon 1 of the Plet1 gene, just downstream of the Kozak sequence but upstream of the ATG. Animal experiments were approved by local Animal Ethics Committees, and performed in accordance with institutional guidelines. Age‐ and gender‐matched littermates were used whenever possible.

### Antibodies

Monoclonal antibodies used were CD11c (HL3; BD), CD103 (2E7; BioLegend), CD11b (M1/70; eBioscience), CD64 (X54‐5/7.1; BioLegend), MHC class II (M5/114.15.2; eBioscience), CD45 (30‐F11; BioLegend), CD45R (RA3‐6B2; BD), CD8α (53‐6.7; eBioscience), CD197 (4B12; eBioscience), Plet1 (1D4), CD38 (90; eBioscience), CD19 (1D3; eBioscience), CD95 (Jo2; BD), Donkey anti‐rat IgG (Life Technologies), CD4 (GK1.5; BD), Foxp3 (Fjk‐16s; eBioscience), IL‐10 (JES5‐16E3; eBioscience), IFNγ (XMG1.2; BD), and IL‐17A (TC11‐18H10; BD Pharmingen). Zombie Aqua Fixable viability kit (BioLegend) was used for dead cell exclusion.

### Flow cytometry

Fc receptors were blocked by pre‐incubation with normal mouse serum. All stainings were performed in PBS containing 2% heat‐inactivated fetal calf serum (FCS) at 4°C, except for the CCR7 staining that was performed at 37°C. For intracellular Foxp3, IL‐10, IL‐17A, and IFNγ, cells were stained for extracellular markers, fixed, and permeabilized with Cytofix/Cytoperm solution (BD) before intracellular staining. Cells were analyzed using a FACS LSRII (BD Biosciences) and data was analyzed with FlowJo software (FlowJo, LLC).

### Radiation chimeras

8 week old C57BL/6J mice were irradiated 9 Gy and subsequently reconstituted by i.v. injection of 1–2 × 10^6^ bone marrow cells from either Plet1^+/+^ or Plet1^−/−^ mice. Mice received oral antibiotics for 2 weeks after bone marrow transplantation, and were analyzed 8 weeks after reconstitution.

### Isolation of cells from intestinal lamina propria

Isolated small intestine was opened longitudinally and washed with cold HBSS containing 15 mM Hepes, pH 7.2. Tissues were cut in 1 cm pieces and incubated in HBSS buffer containing 10% FBS, 15 mM HEPES, 5 mM EDTA, and 1 mM DTT, pH 7.2, at 37°C two times for 20 min to remove epithelium and intraepithelial lymphocytes. Tissues were digested with 100 U/mL Collagenase VIII (Sigma‐Aldrich) in RPMI containing 10% FBS, 15 mM HEPES, 100 U/mL P/S, and 1 mM DTT, pH 7.2, at 37°C in a shaker two times for 1 h. Supernatants were passed through a 100 μm cell strainer and washed in cold HBSS. Pellets were suspended in 90% Percoll (GE Healthcare), overlaid with 40% Percoll, and centrifuged at 1800 rpm for 20 min to allow separation of mononuclear cells by density gradient. Interphase was washed and stained with conjugated antibodies. Lamina propria lymphocytes were analyzed by flow cytometry (LSR II; BD), and Plet1^+^ DC were FACS‐sorted as LiveDead^−^CD45^+^B220^−^CD64^−^MHCII^+^CD11c^+^ (FACSAria III; BD) (see Supporting Information Fig. 1 for full gating strategy).

### Generation and in‐vitro stimulation of BMDC

BMDC were generated from bone marrow suspensions harvested from 8 to 12 weeks old C57BL/6, Plet1^−/−^ mice, and littermate controls. Briefly, bone marrow was flushed from femurs and tibias, passed through a 100 μm mesh to remove fibrous tissue, and red blood cells were lysed using IOTEST lysis solution (Beckman Coulter). Cells were cultured at 0.3 × 10^6^ cells/mL in IMDM medium (GIBCO BRL) supplemented with 10% FCS, 2 mM glutamine, 50 μM β2‐mercaptoethanol, 100 IU/mL penicillin, 100 μg/mL streptomycin and 20 ng/mL Granulocyte‐macrophage colony‐stimulating factor (GM–CSF; X63 supernatant) 40. On day 3 and on day 6, fresh GM‐CSF‐containing medium was added. Floating differentiated BMDC were isolated at day 7 and separated from plate‐adherent macrophages, and used for further experiment. BMDC purity was determined by flow cytometry and was consistently over 60%. For in‐vitro stimulations, 1 × 10^6^ BMDC were cultured overnight in the presence of 5 ng/mL lipopolysaccharide (LPS, Invivogen), or 10 ng/mL Imiquimod (Invivogen).

### Trans‐well migration assay

5 μm pore size trans‐well plates (Corning Costar) were used. Upper chambers were loaded with 5 × 10^4^ BMDC, or FACS‐sorted CD11c^+^MHCII^+^ SI‐LP DC in 200 μL of IMDM supplemented with 10% FBS and 20 ng/mL recombinant murine GM–CSF (cIMDM), and the lower chambers with 500 μL of cIMDM with or without 50 ng/mL recombinant murine CCL19 (R&D Systems). To assess cell migration through 3D collagen, the upper chamber was loaded with 5 × 10^4^ BMDC or sorted CD11c^+^MHCII^+^ SI‐LP DC in cIMDM mixed 1:2 with Collagen type I (Millipore) supplemented with MEM 10X and NaHCO_3_ to pH 7.2. Final concentration of Collagen type I was 3 mg/mL. Following incubation for 2 h, migrated cells were isolated from the bottom compartment and quantified by flow cytometry using Flow Count Fluorospheres (Beckman Coulter).

### IgA ELISA

Fecal pellets were stored in pre‐weighed collection tubes, snap frozen in liquid nitrogen, and kept at ‐80°C until extraction. Samples were resuspended in PBS supplemented with protease inhibitor cocktail (Roche), and capture enzyme‐linked immunosorbent assay (ELISA; Ready‐Set‐Go!, eBioscience) was used to quantify total concentrations of IgA in feces corrected to weight. Mice used for fecal sampling were segregated by genotype after weaning.

### Transcript analysis

RNA was extracted using the NucleoSpin RNA XS kit (Machery‐Nagel). For quantitative PCR (qPCR), a Neviti Thermal Cycler (Applied Biosystems) and DyNAmo Flash SYBR Green qPCR kit (Finnzymes) were used, with the addition of MgCl_2_ to a final concentration of 4 mM. All reactions were performed in duplicate and were normalized to the expression of Gapdh. Relative expression was calculated by the cycling threshold (CT) method as ‐2^ΔCT^. The primer sequences can be found in Supporting Information Table 1.

### RNA sequencing

cDNA was synthesized and amplified using SMARTer Ultra Low RNA kit (Clontech Laboratories) following the manufacturer's protocol. Amplified cDNA was further processed according to TruSeq Sample Preparation v.2 Guide (Illumina) and paired end‐sequenced (2 × 75 base pairs) on the HiSeq 2500 (Illumina). Demultiplexing was performed using CASAVA software (Illumina) and the adaptor sequences were trimmed with Cutadapt (http://code.google.com/p/cutadapt/). Alignments against the mouse genome (mm10) and analysis of differential expressed genes were performed as previously described 41. Cufflinks software was used to calculate the number of fragments per kilobase of exon per million fragments mapped (FPKM) for each gene. FPKM values of Plet1^−/−^ and Plet1^+/+^ DC were then compared to the curated gene sets (C2) and the Gene Ontology gene sets (C7) of the Molecular Signature Database (MSigDB) by GSEA 42 (Broad Institute), using the Signal2Noise metric and 1000 phenotype‐based permutations.

### Histology and immunohistochemistry

For histology, small intestinal tissue pieces were fixed in 4% paraformaldehyde 4 h at room temperature, washed in 70% ethanol and embedded in paraffin. Four‐micrometer sections were deparaffinized and stained with hematoxylin (Vector Laboratories) and eosin (Sigma‐Aldrich). For CD103 and CD3 detection, endogenous peroxidases were blocked in 3% periodic acid in deionized water for 10 min, and antigen retrieval was achieved by microwave treatment in Citric Acid Based citrate buffer (pH 6.0). Prior to staining, Fc receptors were blocked in 10% normal mouse serum and 10% normal goat serum and Armenian hamster serum, 10 mM Tris buffer, 5 mM EDTA, 0.15 M NaCl, 0.25% gelatin, and 0.05% Tween‐20 (pH 8.0). Tissue sections were incubated overnight at 4°C with primary antibodies (CD103: clone 2E7, Biolegend; CD3: Polyclonal A0452, DAKO) in PBS supplemented with 2% normal mouse serum. For immunohistochemistry, tissues were frozen in Tissue‐Tek O.C.T compound (Sakura Finetek Europe B.V.) and stored at ‐80°C. Six μm sections cryosections were fixed for 5 min in ice‐cold aceton and air‐dried for an additional 10min, and subsequently blocked with 5% normal mouse serum and 5% normal donkey serum for 15min. Sections were incubated with primary antibody Rat anti Plet‐1 (1D4) and biotinylated Hamster anti CD11c (N418, Ebioscience) for 1 h at room temperature, followed by a 30‐min incubation with secondary donkey anti‐rat IgG labeled with AlexaFluor‐488 and strepdavidin labeled with AlexaFluor‐594 (Molecular Probes). Sections were embedded in Pro‐long Gold with DAPI (Invitrogen) and analyzed on a Leica DMRXA.

### Statistical analysis

All analyses were performed in GraphPad Prism TM 7 software. Statistical analyses were performed using unpaired Mann–Whitney test. *p* values < 0.05 were considered significant. Data are shown as mean ± SEM.

Venn diagrams were generated using Venny v2.1, (bioinfogp.cnb.csic.es/tools/venny)

## Conflict of interest

The authors declare no commercial or financial conflict of interest.

**Abbreviations**
BMDC: bone marrow‐derived dendritic cell
CCR7: C‐C motif chemokine receptor 7
CT: cycling threshold
DC: dendritic cell
GPI: glycophoshatidylinositol
GSEA: gene set enrichment analysis
LN: lymph node
mAb: monoclonal antibody
MLN: mesenteric lymph node
Plet1: placenta‐expressed transcript 1
qPCR: quantitative PCR
SI‐LP: small intestine lamina propria

## Supporting information

Peer review correspondence

Table 1: list of primer sequences used for RT‐PCR
Table 2. Genes overexpressed in CD103+CD11b+ intestinal DC and induced after colonization.
Table 3: List of differentially expressed genes in Plet1‐/‐ CD11b+CD103+ DCs versus Plet1+/− CD11b+CD103+ DCs
Figure 1: Gating strategy for small intestinal DCs. representative FACS plots of total Lamina propria, describing the gating strategy used in this study to isolate different murine DC subsets.
Figure 2: Plet1 is absent from macrophages and monocytes. representative FACS plots showing absence of Plet1 on CD64+ monocytes and macrophages in Lamina Propria of WT mice.
Figure 3: Plet1‐deficiency does not affect BMDC development or activation. (A) Frequency of CD11c+MHCII+ BMDC after seven days of culture, using Plet1+/− or Plet1‐/‐ bone marrow. (B) Frequency of activated Plet1‐/‐ BMDC, as shown in A, expressing the costimulatory molecules CD40, or CD86, as compared to littermate controls, quantified by flow cytometry. (C) Surface expression of costimulatory molecules (CD40, CD80, CD83, and CD86) on Plet1+/− (black bars), or Plet1‐/‐ (grey bars) BMDC, following TLR4, and TLR7 stimulation, shown as mean fluorescence intensity quantified by flow cytometry. (D) Relative transcript levels by QPCR (normalized to GAPDH) of activation‐induced cytokines (IL6, IL1b, IL23, IL12, and TNF) on Plet1‐/‐ BMDC or littermate controls, following culture in the presence or absence of Pam3Cys (TLR3 ligand), LPS (TLR4 ligand), or Imiquimod (TLR7 ligand).
Figure 4: in‐silico 3D structure prediction of Plet1 protein reveals homology with the integrin‐binding domain of Reelin. (A) Ribbon diagram 3D structure representation of murine, and human Plet1 protein and the integrin‐binding N‐terminal domain of Reelin protein as predicted by Phyre 2 (RCSB Protein Databank, structure c3cooB). (B) Alignment of protein sequence of human and mouse Plet1 with human or mouse Reelin.
