# Supplementary material for: Expression of Plet1 controls interstitial migration of murine small intestinal dendritic cells
Source: Eur J Immunol. 2018 Dec 14;49(2):290–301. doi: 10.1002/eji.201847671 (PMC6492104; doi:10.1002/eji.201847671)

European Journal of Immunology

Supporting Information

for

DOI 10.1002/eji.201847671

Julien J. Karrich, Mónica Romera-Hernández, Natalie Papazian, Sharon Veenbergen, Ferry Cornelissen, Patricia Aparicio-Domingo, Frances H. Stenhouse, C. Diana Peddie, Remco M. Hoogenboezem, Chelsea W. J. den Hollander, Terri Gaskell, Tanya Medley, Louis Boon, C. Clare Blackburn, David R. Withers, Janneke N. Samsom and Tom Cupedo

Expression of Plet1 controls interstitial migration of murine small intestinal dendritic cells

© 2018 The Authors. *European Journal of Immunology* published by WILEY-VCH Verlag GmbH & Co. KGaA, Weinheim.

SUPPLEMENTARY TABLES AND FIGURES

Expression of Plet1 controls interstitial migration of murine small intestinal dendritic cells

Julien J. Karrich[1], Mónica Romera-Hernández[1], Natalie Papazian[1], Sharon Veenbergen[2], Ferry Cornelissen[1], Patricia Aparicio-Domingo[1,*], Frances H. Stenhouse[3], C. Diana Peddie[3], Remco M. Hoogenboezem[1], Chelsea W. J. den Hollander[1], Terri Gaskell[3,**], Tanya Medley[3,***], Louis Boon[4], C. Claire Blackburn[3], David R. Withers[5], Janneke N. Samsom[2], and Tom Cupedo[1].

Supplementary table 1: list of primer sequences used for RT-PCR

Plet1	Forward	5'-CGTGGTCCTTGATAACATCT-3'
Plet1	Reverse	5'-TCACGGCACTGACTGAA-3'
IL-12P40	Forward	5'-TGGCTGGTGCAAAGAA-3'
IL-12P40	Reverse	5'-GGGTCTGGTTTGATGATGT-3'
IL1β	Forward	5'-GGACCCCAAAAGATGAAG-3'
IL1β	Reverse	5'-CCACGGGAAAGACACAG-3'
Il-23p19	Forward	5'-GCCCCGTATCCAGTGT-3'
Il-23p19	Reverse	5'-CTGCCACTGCTGACTAGAA-3'
IL-6	Forward	5'-TCGGAGGCTTAATTACACA-3'
IL-6	Reverse	5'-CTGGCTTTGTCTTTCTTGTT-3'
Gapdh	Forward	5'-TCAACGGCACAGTCAAG-3'
Gapdh	Reverse	5'-GCTCCACCCTTCAAGTG-3'
TNF	Forward	5'-GGGGGCTTCCAGAACT-3'
TNF	Reverse	5'-GGGCCATAGAACTGATGAG-3'

Supplementary Table 2. Genes overexpressed in CD103+CD11b+ intestinal DC and induced after colonization.

Gene Symbol	Gene Title	Protein Class and Function
Trim72	Tripartite Motif Containing 72	
Clec4a3	C-type lectin domain family 4, member a3	Immunoglobulin receptor superfamily
Gbp3	Guanylate Binding Protein 3	Heterotrimeric G-protein
Gbp7	Guanylate Binding Protein 7	Heterotrimeric G-protein
Vwa5a	Von Willebrand Factor A Domain Containing 5A	Serine protease inhibitor
Cd86	Cluster of Differentiation 86	T-lymphocyte activation antigen
**Plet1**	**Placenta Expressed Transcript 1**	**Cell migration and cellular adhesion to matrix proteins[14]**
Tha1	Threonine Aldolase	Aldolase
Ifitm3	Interferon Induced Transmembrane Protein 3	
Gbp8	Guanylate Binding Protein 3	Heterotrimeric G-protein
H2-Eb1	histocompatibility 2, class II antigen E beta	Major histocompatibility complex antigen
Tmem171	Transmembrane Protein 171	
Rtp4	Receptor Transporter Protein 4	Olfactory receptor binding
Blm	Bloom Syndrome RecQ Like Helicase	DNA helicase
Gm4759	GTPase, very large interferon inducible 1 pseudogene	
**Lgals9**	**Galectin 9**	**Cell adhesion molecule, signaling molecule[43]**
Pgs1	Phosphatidylglycerophosphate Synthase 1	Cytokine, extracellular matrix protein, receptor
Spi1	Transcription Factor PU.1	Nucleic acid binding signaling molecule transcription factor
**Celsr1**	**Cadherin, EGF LAG Seven-Pass G-Type Receptor 1**	**G-protein coupled receptor cadherin, cell adhesion[44]**
Ltf	Lactotransferrin	Serine transferase/carrier protein

Supplementary Table 3: List of differentially expressed genes in Plet1$^{-/-}$ CD11b^{+}CD103^{+} DCs versus Plet1$^{+/-}$ CD11b^{+}CD103^{+} DCs.

	base mean	log2 fold change	p value
Zg16	295,7178293	3,803648649	1,01E-29
Clca3	253,6791646	3,666473016	2,22E-26
Reg1	324,7811166	3,581519814	1,53E-21
Fcgbp	317,5764988	3,394766698	4,53E-20
Gpx3	175,4673748	3,313596713	1,69E-17
Reg3g	177,777684	3,304552688	1,04E-18
Sparc	151,9958387	3,124838317	3,24E-15
Spink4	144,0538515	2,82444654	5,62E-12
Muc13	151,0492757	2,520111199	2,47E-10
Mgst1	97,71867738	2,325635251	2,90E-08
Slc12a2	158,0932112	2,206663867	5,78E-09
Igj	2862,760185	2,201395662	1,11E-06
Krt18	291,7483196	2,195756014	1,19E-09
Cldn7	181,643058	2,164610628	4,94E-08
Muc2	212,5394136	2,158785949	9,34E-10
Krt19	490,8249061	2,144642548	2,92E-09
Gas6	74,73268984	2,06601817	2,94E-06
Ppp1r1b	77,20610416	2,054836619	3,10E-06
Lgals2	441,7932377	2,033738434	3,10E-08
Mttp	158,4624469	2,014767214	3,91E-06
Kcnn4	63,56646777	1,974365102	1,18E-05
Atp1b1	119,0737894	1,951990887	3,07E-06
Guca2a	76,92997669	1,951740744	1,25E-05
Prap1	204,3643923	1,951191601	4,26E-08
Lgals4	299,1707807	1,905245488	5,56E-08
Sdc1	53,51943065	1,904108717	2,56E-05
Cyr61	138,7789374	1,898323788	4,08E-07
Dcn	426,5102989	1,844637034	3,70E-05
Aldob	274,8394836	1,825978801	1,73E-06
Ces2e	104,1818783	1,814291578	4,33E-05
Lypd8	261,0998182	1,794928927	8,05E-06
Gpx2	105,359648	1,784097125	4,13E-05
Krt7	100,1245896	1,780515681	3,64E-05
Adh6a	69,84324206	1,769436703	5,27E-05
Papss2	85,86906265	1,745778184	0,00010075
Klf5	118,1354687	1,721421536	5,96E-05
Ep1	73,43456377	1,696634177	0,00015123
Fabp1	187,8102662	1,688513312	2,92E-05
Fam84b	89,34915787	1,665026519	0,00011788
Tmc4	82,58530517	1,645956974	0,00012863
Klf2	206,2340749	1,643823934	4,85E-05
Cd81	182,9001769	1,618630249	3,83E-06
Nusap1	160,7075052	1,60856318	1,53E-05
Myo1a	161,1085243	1,592656852	0,0002262
Sema4a	96,41458019	1,564883544	0,00017622
Alpi	210,8479608	1,559413192	6,81E-05
Id3	197,1149588	1,53002372	3,76E-05
Smc2	183,5783059	1,521078056	1,49E-05
Mapk13	129,5006959	1,515005186	0,00010016
Elf3	217,7544976	1,500207478	6,72E-05
Tmem176b	147,9234055	1,471606326	0,00030006
Fabp2	359,0487168	1,444147602	0,00025921
Phlda1	313,159568	1,327781391	0,00019923
Dnaja4	498,9556356	1,222627282	0,00017997
Golm1	370,2671965	1,134474633	0,00014362
Dnajb1	12036,73036	1,036998264	1,94E-05
Hspd1	6512,07026	0,98247914	9,43E-05
Txnip	1066,518523	0,941460822	5,51E-06
Hspa1b	20320,58879	0,908284873	4,36E-05

Tsc22d3	1636,639014	0,889774248	6,21E-05
Hsp90aa1	13237,02169	0,80967743	5,91E-05
Pts	1988,39691	0,692957305	0,00024314
Mon1b	757,3008838	-0,842326384	0,00023682
Kdm6a	1313,905028	-0,909889833	4,43E-05
Mapkbp1	128,0258742	-1,725894145	2,32E-05
Plet1	5055,29523	-6,679885454	3,52E-284

Supplementary Figure Legends

Supplementary figure 1: Gating strategy for small intestinal DCs. representative FACS plots of total Lamina propria, describing the gating strategy used in this study to isolate different murine DC subsets.

Supplementary figure 2: Plet1 is absent from macrophages and monocytes. representative FACS plots showing absence of Plet1 on CD64^{+} monocytes and macrophages in Lamina Propria of WT mice.

Supplementary figure 3: Plet1-deficiency does not affect BMDC development or activation. (A) Frequency of CD11c^{+}MHCII^{+} BMDC after seven days of culture, using Plet1$^{+/-}$ or Plet1$^{-/-}$ bone marrow. (B) Frequency of activated Plet1$^{-/-}$ BMDC, as shown in A, expressing the costimulatory molecules CD40, or CD86, as compared to littermate controls, quantified by flow cytometry. (C) Surface expression of costimulatory molecules (CD40, CD80, CD83, and CD86) on Plet1$^{+/-}$ (black bars), or Plet1$^{-/-}$ (grey bars) BMDC, following TLR4, and TLR7 stimulation, shown as mean fluorescence intensity quantified by flow cytometry. (D) Relative transcript levels by QPCR (normalized to GAPDH) of activation-induced cytokines (IL6, IL1β, IL23, IL12, and TNF) on Plet1$^{-/-}$ BMDC or littermate controls, following culture in the presence or absence of Pam3Cys (TLR3 ligand), LPS (TLR4 ligand), or Imiquimod (TLR7 ligand).

Supplementary figure 4: *in-silico* 3D structure prediction of Plet1 protein reveals homology with the integrin-binding domain of Reelin. (A) Ribbon diagram 3D structure representation of murine, and human Plet1 protein and the integrin-binding N-terminal domain of Reelin protein as predicted by Phyre 2 (RCSB Protein Databank, structure c3cooB). (B) Alignment of protein sequence of human and mouse Plet1 with human or mouse Reelin.

Supplementary figure 1

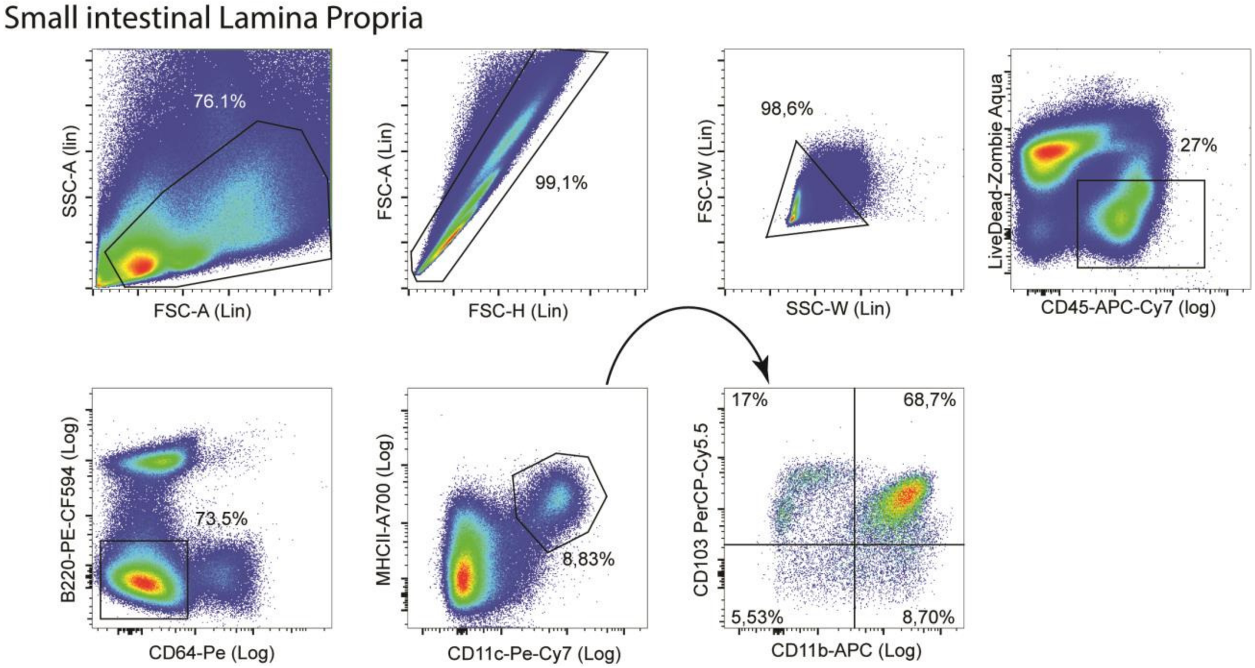

Supplementary figure 2

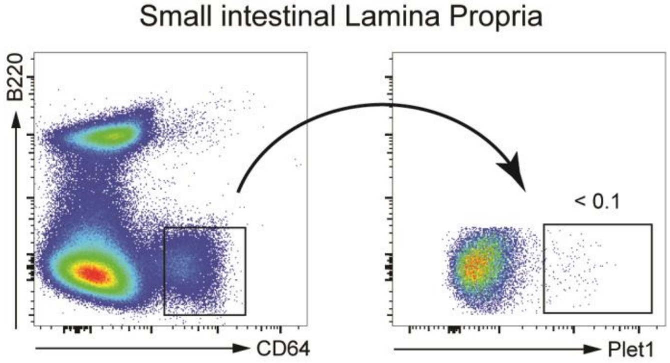

Supplementary figure 3

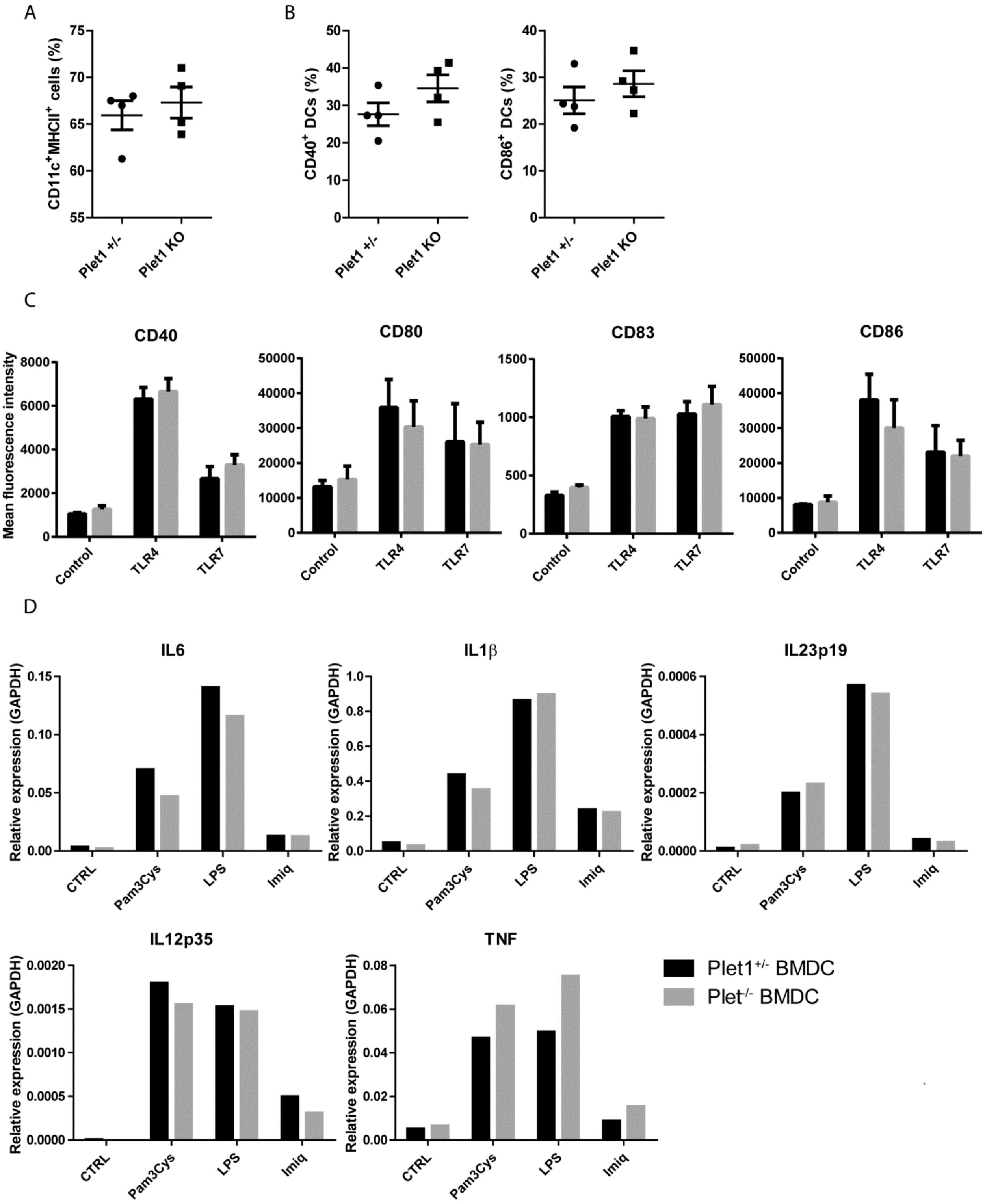

Supplementary figure 4

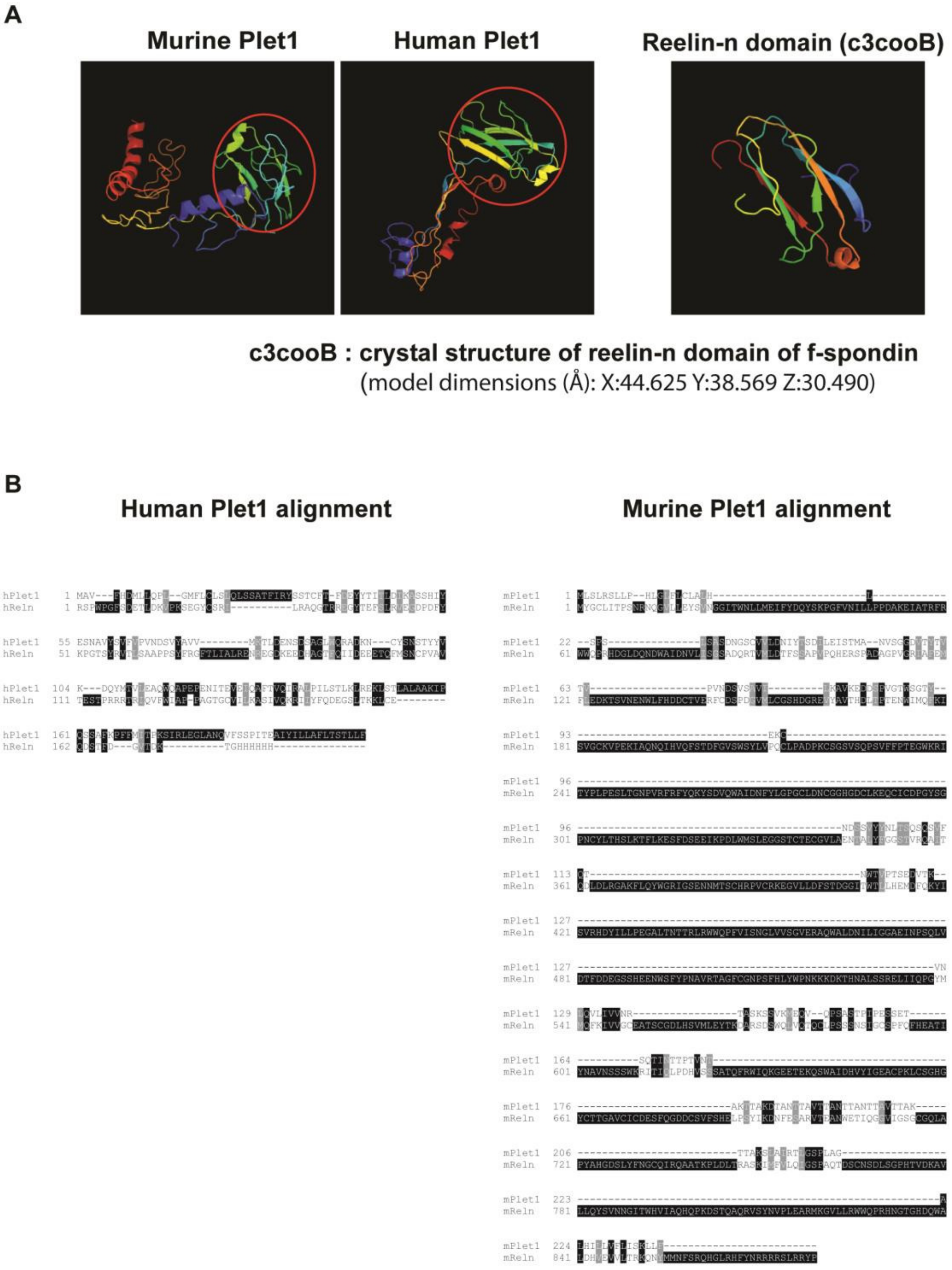

Supplement: Supplementary file 2 — Table 1: list of primer sequences used for RT‐PCR Table 2. Genes overexpressed in CD103+CD11b+ intestinal DC and induced after colonization. Table 3: List of differentially expressed genes in Plet1‐/‐ CD11b+CD103+ DCs versus Plet1+/− CD11b+CD103+ DCs Figure 1: Gating strategy for small intestinal DCs. representative FACS plots of total Lamina propria, describing the gating strategy used in this study to isolate different murine DC subsets. Figure 2: Plet1 is absent from macrophages and monocytes. representative FACS plots showing absence of Plet1 on CD64+ monocytes and macrophages in Lamina Propria of WT mice. Figure 3: Plet1‐deficiency does not affect BMDC development or activation. (A) Frequency of CD11c+MHCII+ BMDC after seven days of culture, using Plet1+/− or Plet1‐/‐ bone marrow. (B) Frequency of activated Plet1‐/‐ BMDC, as shown in A, expressing the costimulatory molecules CD40, or CD86, as compared to littermate controls, quantified by flow cytometry. (C) Surface expression of costimulatory molecules (CD40, CD80, CD83, and CD86) on Plet1+/− (black bars), or Plet1‐/‐ (grey bars) BMDC, following TLR4, and TLR7 stimulation, shown as mean fluorescence intensity quantified by flow cytometry. (D) Relative transcript levels by QPCR (normalized to GAPDH) of activation‐induced cytokines (IL6, IL1b, IL23, IL12, and TNF) on Plet1‐/‐ BMDC or littermate controls, following culture in the presence or absence of Pam3Cys (TLR3 ligand), LPS (TLR4 ligand), or Imiquimod (TLR7 ligand). Figure 4: in‐silico 3D structure prediction of Plet1 protein reveals homology with the integrin‐binding domain of Reelin. (A) Ribbon diagram 3D structure representation of murine, and human Plet1 protein and the integrin‐binding N‐terminal domain of Reelin protein as predicted by Phyre 2 (RCSB Protein Databank, structure c3cooB). (B) Alignment of protein sequence of human and mouse Plet1 with human or mouse Reelin. [file EJI-49-290-s002.pdf]
