# Supplementary material for: Expression of Plet1 controls interstitial migration of murine small intestinal dendritic cells
Source: Eur J Immunol. 2018 Dec 14;49(2):290–301. doi: 10.1002/eji.201847671 (PMC6492104; doi:10.1002/eji.201847671)
Supplement: Supplementary file 1 — Peer review correspondence [file EJI-49-290-s001.pdf]

European Journal of Immunology

Expression of Plet1 controls interstitial migration of murine small intestinal dendritic cells

---

Review Timeline:	Submission date:	30-Apr-2018
	First Editorial decision:	20-Jun-2018
	Revision received:	30-Oct-2018
	Accepted:	5-Dec-208

Handling Executive Committee member: Prof. Kenneth Murphy

Please note that the correspondence below does not include the standard editorial instructions regarding preparation and submission of revised manuscripts, only the scientific revisions requested and addressed.

First Editorial Decision
20-Jun-2018

Dear Dr. Cupedo,

Manuscript ID eji.201847671 entitled "Microbiota-regulated Expression of Plet1 Controls Interstitial Migration of Small Intestinal Dendritic Cells", which you submitted to the European Journal of Immunology, has been reviewed. The comments of the referees are included at the bottom of this letter. We are sorry for the delay in final decision, we've had series of circumstances that led to this, namely, one of the referees were delayed, and our Executive Committee and internal editorial attending a conference.

Although the referees have recommended publication, some revisions to your manuscript have been requested. Therefore, I invite you to respond to the comments of the referees and revise your manuscript accordingly. You will note that Referee 2 suggested an experiment that would "test the impact of the altered DC migration on immune responses". Since this referee was not specific in his request, we will leave you with the option of performing an experiment with tools/models available in your lab.

You should also pay close attention to the editorial comments included below. **In particular, please edit your figure legends to follow Journal standards as outlined in the editorial comments. It seems that most of the data shown are from three experiments, but the number of mice (or samples) per experiment was not stated. Please also show gating strategy, fluorochrome axis labels and percent of cells within a gate for all flow cytometry data. Failure to do this will result in delays in the re-review process.**

If the revision of the paper is expected to take more than three months, please inform the editorial office. Revisions taking longer than six months may be assessed by new referees to ensure the relevance and timeliness of the data.

Once again, thank you for submitting your manuscript to European Journal of Immunology. We look forward to receiving your revision.

Yours sincerely,
Nadja Bakocevic

on behalf of
Prof. Kenneth Murphy

Dr. Nadja Bakocevic
Editorial Office
European Journal of Immunology
e-mail: ejied@wiley.com

www.eji-journal.eu

****

Reviewer: 1

Comments to the Author
In the paper entitled â€˜Microbiota-regulated expression of Plet1 controls interstitial migration of small intestinal dendritic cellsâ€™, Karrich et al. demonstrated that PLET1 induced by intestinal microbiota is required for dendritic cell (DC) migration. Authors also present the transcriptome results suggesting that PLET1 represses the interaction of DCs with extra cellular matrix (ECM). Findings presented in this manuscript is novel, and beneficial for immunologists to understand molecular mechanisms of DC migration, but there are concerns to be addressed.

1. Plet1-deficient DCs do not migrate from the small intestine to mesenteric lymph node. However, it is unclear whether Plet1-deficient DCs do not move at all, or they migrate and accumulate at particular areas where is potentially rich for ECM. DC localization or motility should be tested by histology or intravital microscopy to understand the migratory characteristics of Plet1-deficient DCs.

2. Page 3, line 37. that microbial signals control
Since there is no description about the association between microbiota and Plet1 prior to this sentence, â€˜microbial signalsâ€™ only in conclusion seems to be abrupt.

3. Figure 4D seems to be missing, and current Figure 4D is supposed be Figure 4E. The proper results should be added, and the figure should be corrected.

4. Letters in Figure 5C are too small, and hard to read.

5. Magnifications of diagrams in Supplementary Figure 4 are different among proteins, and hard to compare. Also, amino acid sequence besides the 3D diagram could be helpful to compare their similarity.

6. Since FLT3L-induced BMDCs are more sensitive to CCR7-ligand chemokines than GM-CSF-induced BMDCs (J Immunol 193: 4904), the former cells could be more useful to test the function of PLET1 in DC migration in future studies.

Reviewer: 2

Comments to the Author
Karrich and colleagues have submitted a well-written and thoughtful paper related to the role of Plet1 and DC migration through the extracellular matrix, independent of CCR7 function. The experiments appear to have been properly and the figures are clear. My only suggestions are as follows;
1) The authors to not describe any experiments designed to test the impact of the altered DC migration on immune responses. As dendritic cells are key players in the initiation of immune responses, such an experiment would strengthen the paper considerably.
2) It is unclear where, exactly, the increased number of double positive DCs in the small intestinal lamina propria reside. Immunohistochemistry might provide helpful and interesting information in this regard, although it is probably not essential to the paper.

<u>First Revision – authors' response</u>

<u>30-Oct-2018</u>

We are grateful to the reviewers for their positive comments and constructive criticism on our manuscript, and the opportunity to revise and improve our study. In response to the reviewers

comments we have included new experiments and altered text and figures of the manuscript. Please find a detailed point-by-point reply below.

Reviewer 1

1. Plet1-deficient DCs do not migrate from the small intestine to mesenteric lymph node. However, it is unclear whether Plet1-deficient DCs do not move at all, or they migrate and accumulate at particular areas where is potentially rich for ECM. DC localization or motility should be tested by histology or intravital microscopy to understand the migratory characteristics of Plet1-deficient DCs.

The reviewer raises an interesting point, that was also mentioned by reviewer 2. To address the question of DC localization in the intestine we labeled small intestinal sections from Plet1+/- and Plet1-/- mice with CD103 and CD3. This allowed us to visualize CD103 expressing T cells and CD103 expressing (CD3 negative) DCs. In line with our flow cytometric analyses, we identified villi that appeared to contain a higher number of CD103+ DC in Plet1-/- mice compared to the heterozygous controls. Even though this was consistently seen in 5 vs 5 mice, the distribution throughout the small intestine was not uniform. While some villi appeared to have a higher number of DC, other villi in the same section did not show this difference. In terms of location, the increased number of CD103+ DCs seemed mostly located to the bottom half of the villus. At this moment it is unclear whether this might correlate with altered composition of the ECM at these locations. We have added representative histological images as new Figure 4A, showing villi with normal and increased numbers of DC. Because of this heterogeneity we believe that the quantification by flow cytometry as in Figures 4B-C is still the most truthful representation of the differences now visualized in Figure 4A.

2. Page 3, line 37. '… that microbial signals control…'
Since there is no description about the association between microbiota and Plet1 prior to this sentence, 'microbial signals' only in conclusion seems to be abrupt.

We agree with the reviewer that this sentence disrupted the flow of the abstract and it has therefore been removed. For this same reason the microbiota reference has also been removed from the manuscript title.

3. Figure 4D seems to be missing, and current Figure 4D is supposed be Figure 4E. The proper results should be added, and the figure should be corrected.

We apologize for this erroneous inclusion of the incorrect figure during final submission. Figure 4 has been replaced with the correct figure that includes panel 4D and now fully aligns with the text.

4.Letters in Figure 5C are too small, and hard to read.

We have re-made the panels in figure 5C to increase font size and general readability.

5. Magnifications of diagrams in Supplementary Figure 4 are different among proteins, and hard to compare. Also, amino acid sequence besides the 3D diagram could be helpful to compare their similarity.

We thank the reviewer for pointing out this issue. We have replaced the diagrams in Supplemental figure 4A and have added protein sequence alignments of mouse Plet1-Reelin and human Plet1-Reelinin supplemental figure 4B.

6. Since FLT3L-induced BMDCs are more sensitive to CCR7-ligand chemokines than GM-CSF-induced BMDCs (J Immunol 193: 4904), the former cells could be more useful to test the function of PLET1 in DC migration in future studies.

The reviewer raises an important point relevant to most studies using in vitro generated dendritic cells. multiple methods have been described to generate in vitro DCs, yet a definitive consensus on which culture method is appropriate in individual cases remains to be established. The reviewer is right in pointing out that Flt3l-induced DC could have different, or most likely even better CCR7 responses in our in vitro 3D culture system. We have added the following text to the discussion to highlight this fact:

"Our in-vitro experiments were performed with isolated lamina propria DC as well as with GM-CSF matured BMDC. It is important to realize that expression of CCR7 on BMDC is lower in GM-CSF-derived cultures compared to FLT3L-matured BMDC [32]. This might imply that the differences observed could be even more pronounced when using alternative methods of cell preparation."

Reviewer 2

1. The authors to not describe any experiments designed to test the impact of the altered DC migration on immune responses. As dendritic cells are key players in the initiation of immune responses, such an experiment would strengthen the paper considerably.

We agree with the reviewer that linking functional alterations in downstream immune responses with absence of Plet1 expression would have been of added value to our study. We show in our study that steady state, ex-vivo, interstitial migration is reduced when DCs lack Plet1 expression. To define whether this would lead to alterations in immune parameters at steady state we evaluated immune cell composition and IgA production as shown in the original Figure 3. Currently, using the models available in our lab, we have not been able to identify alterations in immune responses after challenge. Oral gavage of the TLR7/8 ligand R848 induces egress of DCs from the lamina propria into the gut-draining mesenteric LN (MLN), both in Plet1+/- controls and in Plet1-/- animals (shown below as reviewer figure 1a). This suggests that Plet1-deficiency does not impair migration of activated DC, and if anything, a trend towards a higher percentage of CD11b+CD103+ DC in the MLN is seen. On the other hand, the DC distribution within the few remaining intestinal DCs is not altered (reviewer figure 1b), suggesting that the trend towards more CD103+CD11b+ DC in the lymph nodes likely reflects the presence of more of these cells in the intestine prior to activation. Differential regulation of migration by Plet1 in activated vs non-activated DC could be of interest, but goes beyond the scope of the current study.

To test whether Plet1 deficiency would alter pathology after small intestinal damage, we exposed Plet1-/- mice and littermate controls to Methotrexate, a model we have previously used to study tissue damage and repair (Aparicio-Domingo et al., J. Exp. Med. 2015 Oct 19;212(11):1783-91). At day 4 after the final MTX injection, no differences in intestinal pathology were found between the two groups (reviewer figure 1c), indicating no major effect of Plet1 absence on tissue repair in this model.

In sum we conclude that Plet1-mediated regulation of migration might be altered by (strong) activation of DCs, and that with the models presently available to us we are unable to define differences in pathology in the absence of Plet1. The limited scope of our model and the possible loss of Plet1 effects after activation make that we are not able to draw any definitive conclusions from these observations, that will require in-depth analyses in future studies. For these reasons we have not included these preliminary experiments in the manuscript but show them below for the reviewers discretion only.

2. It is unclear where, exactly, the increased number of double positive DCs in the small intestinal lamina propria reside. Immunohistochemistry might provide helpful and interesting information in this regard, although it is probably not essential to the paper.

The reviewer raises an interesting point, that was also mentioned by reviewer 1. To address the question of DC localization in the intestine we labeled small intestinal sections from Plet1+/- and Plet1-/- mice with CD103 and CD3. This allowed us to visualize CD103 expressing T cells and CD103 expressing (CD3 negative) DCs. In line with our flow cytometric analyses, we identified villi that appeared to contain a higher number of CD103+ DC in Plet1-/- mice compared to the heterozygous controls. Even though this was consistently seen in 5 vs 5 mice, the distribution throughout the small intestine was not uniform. While some villi appeared to have a higher number of DC, other villi in the same section did not show this difference. In terms of location, the increased number of CD103+ DCs seemed mostly located to the bottom half of the villus. At this moment it is unclear whether this might correlate with altered composition of the ECM at these locations. We have added representative histological images as new Figure 4A, showing villi with normal and increased numbers of DC. Because of this heterogeneity we believe that the quantification by flow cytometry as in Figures 4B-C is still the most truthful representation of the differences now visualized in Figure 4A.

European Journal of Immunology

Reviewer Figure 1

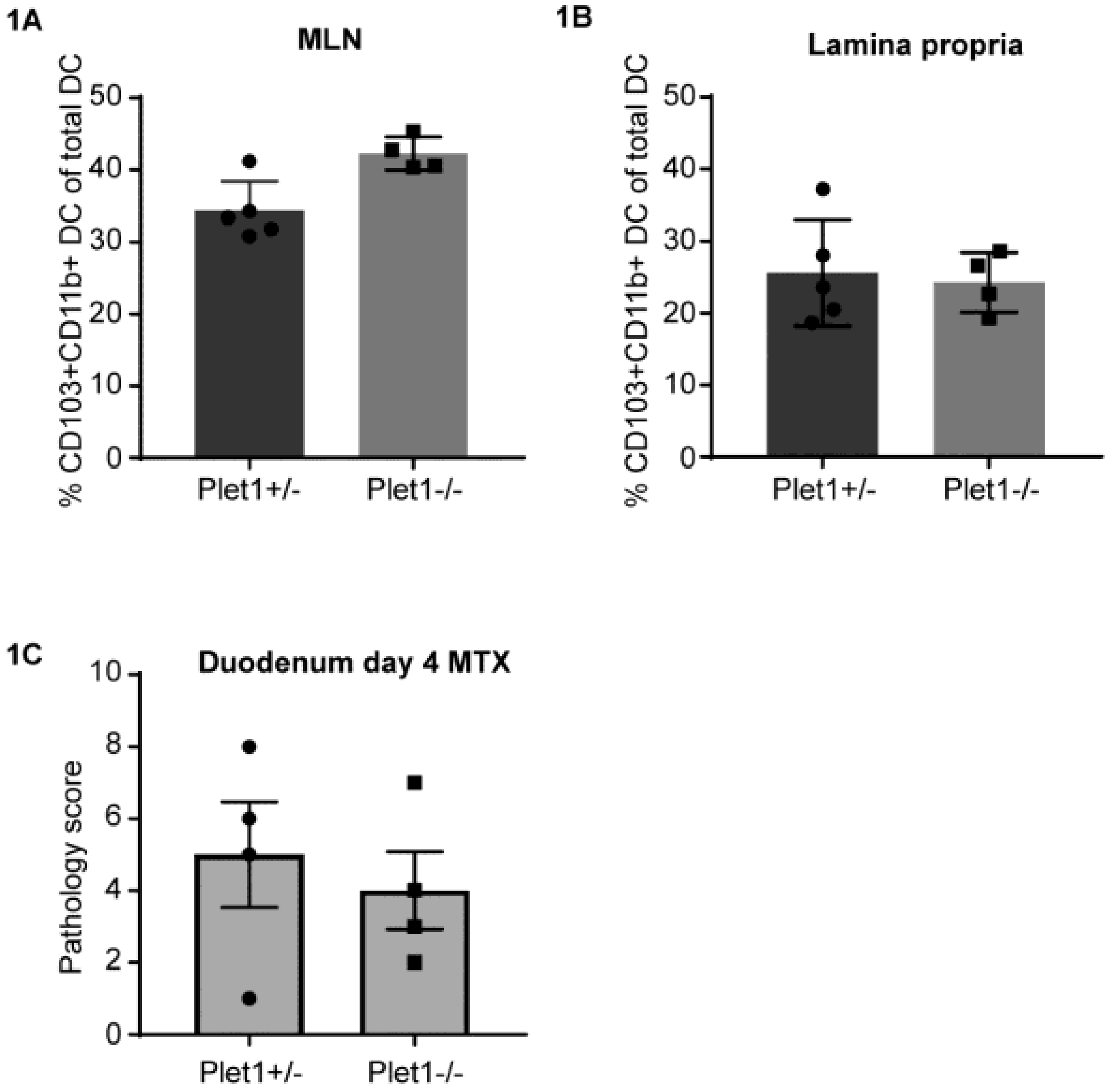

Second Editorial Decision

21-Nov-2018

Dear Dr. Cupedo,

It is a pleasure to provisionally accept your manuscript entitled "Expression of Plet1 Controls Interstitial Migration of Small Intestinal Dendritic Cells" for publication in the European Journal of Immunology. For final acceptance, please follow the instructions below and return the requested items as soon as possible as we cannot process your manuscript further until all items listed below are dealt with.

Please note that EJI articles are now published online a few days after final acceptance (see Accepted Articles: https://onlinelibrary.wiley.com/toc/15214141/0/ja). The files used for the Accepted Articles are the final files and information supplied by you in Manuscript Central. You should therefore check that all the information (including author names) is correct as changes will NOT be permitted until the proofs stage.

We look forward to hearing from you and thank you for submitting your manuscript to the European Journal of Immunology.

Yours sincerely,
Nadja Bakocevic

on behalf of
Prof. Kenneth Murphy

Dr. Nadja Bakocevic
Editorial Office
European Journal of Immunology
e-mail: ejied@wiley.com
www.eji-journal.eu
